# Polymorphisms in *MDM2* and *TP53* Genes and Risk of Developing Therapy-Related Myeloid Neoplasms

**DOI:** 10.1038/s41598-018-36931-x

**Published:** 2019-01-17

**Authors:** Maria Cabezas, Lydia García-Quevedo, Cintia Alonso, Marta Manubens, Yolanda Álvarez, Joan Francesc Barquinero, Santiago Ramón y Cajal, Margarita Ortega, Adoración Blanco, María Rosa Caballín, Gemma Armengol

**Affiliations:** 1 Unit of Biological Anthropology, Department of Animal Biology, Plant Biology and Ecology, Faculty of Biosciences, Universitat Autònoma de Barcelona, 08193 Bellaterra, Catalonia Spain; 2 Department of Pathology, Vall d’Hebron University Hospital, 08035 Barcelona, Catalonia Spain; 3 Spanish Biomedical Research Network Centre in Oncology (CIBERONC), Barcelona, Catalonia Spain; 4 Department of Hematology, Vall d’Hebron University Hospital, 08035 Barcelona, Catalonia Spain

## Abstract

One of the most severe complications after successful cancer therapy is the development of therapy-related myeloid neoplasms (t-MN). Constitutional genetic variation is likely to impact on t-MN risk. We aimed to evaluate if polymorphisms in the p53 pathway can be useful for predicting t-MN susceptibility. First, an association study revealed that the Pro variant of the *TP53* Arg72Pro polymorphism and the G allele of the *MDM2* SNP309 were associated with t-MN risk. The Arg variant of *TP53* is more efficient at inducing apoptosis, whereas the Pro variant is a more potent inductor of cell cycle arrest and DNA repair. As regards *MDM2* SNP309, the G allele is associated with attenuation of the p53 apoptotic response. Second, to evaluate the biological effect of the *TP53* polymorphism, we established Jurkat isogenic cell lines expressing p53Arg or p53Pro. Jurkat p53Arg cells presented higher DNA damage and higher apoptotic potential than p53Pro cells, after treatment with chemotherapy agents. Only p53Pro cells presented t(15;17) translocation and del(5q). We suggest that failure to repair DNA lesions in p53Arg cells would lead them to apoptosis, whereas some p53Pro cells, prone to cell cycle arrest and DNA repair, could undergo misrepair, generating chromosomal abnormalities typical of t-MN.

## Introduction

Therapy-related myeloid neoplasms (t-MN) include therapy-related acute myeloid leukemia (t-AML) and therapy-related myelodysplastic syndrome (t-MDS), which in most cases will progress to overt AML. They are neoplastic hematopoietic disorders occurring as a late complication after chemotherapy and/or radiation therapy used to treat other malignancies and are recognized as a distinct entity by the World Health Organization classification^1^. T-MN constitute the most frequent secondary neoplasias, and their incidence has risen dramatically over the last few years, as both the number of patients receiving cytotoxic agents and the population of long-term cancer survivors have increased^2^. Collected data suggest that up to 20% of patients treated for a primary cancer develop t-MN^3^ with latency periods between diagnosis of the primary disease and occurrence of t-MN ranging between several months to several years^4^. Patients with t-MN are typically resistant to conventional treatment and are considered to have an inferior outcome compared with *de novo* MN^5^.

Cytotoxic agents, like alkylating agents and topoisomerase II poisons in addition to radiotherapy, are associated with t-MN. Depending on the therapeutic agent used to treat the primary cancer, two types of t-AML can be distinguished. The most common, comprising 75–90% of cases, occurs 5 to 10 years after exposure to alkylating agents or radiation, and is frequently accompanied by clonal unbalanced cytogenetic abnormalities, such as the loss of part or all of chromosomes 5 or 7 or both^4,6^. It is preceded frequently by MDS and mutations of the *TP53* tumor suppressor gene are also common^7^. The less common subtype arises after treatment with drugs targeting topoisomerase II. It is characterized by a typical latency of only 1 to 5 years, previous MDS is rare, and it harbors balanced rearrangements involving *MLL* at 11q23 or less often *RUNX1* at 21q22 or t(15;17) *PML*-*RARA*^4,8,9^.

Apart from killing tumoral cells, alkylating agents and topoisomerase II poisons can also induce high levels of DNA damage (mainly double strand breaks, DSBs) in rapidly dividing non-tumoral cells, such as hematopoietic progenitor cells. These DSBs can be repaired by homologous recombination repair (HRR) or non-homologous end joining (NHEJ). However, misrepair of these highly mutagenic lesions can lead to chromosomal alterations and genomic instability that can induce leukemic transformation of cells and can ultimately give rise to t-MN, if accompanied by inappropriate avoidance of cell death. Otherwise, failure to repair DNA DSB, if DNA damage is irreversible, results in an apoptotic response and cell death (reviewed in^10^). Interestingly, there is evidence of large inter-individual variations in DNA repair capacity, which may be due to differences in genetic makeup^11^. In this regard, a good constitutive capacity for DSB repair and/or apoptosis could be crucial for avoiding malignant stem cell transformation and leukemogenesis associated with the clinical use of some cytotoxic drugs. Noteworthy, only a subset of all patients exposed to genotoxic therapy develop t-MN. Moreover, individuals who develop a t-MN are at particularly high risk of developing multiple subsequent cancers^12^. Overall, these data suggest that genetic factors may contribute to t-MN risk. Studying the heritable predisposition to t-MN is of paramount importance, since the identification of patients prone to develop it would enable planning treatment protocols designed to minimize risk. Not surprisingly, several studies have identified polymorphisms associated with increased t-MN susceptibility in a number of genes involved in drug metabolism or DNA repair (reviewed by^13^). However, most of these studies have produced inconclusive results. This might be due in part because many of these works used healthy individuals or *de novo* MN samples as a reference group, instead of patients who had undergone therapy for a primary tumor but had not developed t-MN some years later. So far, five studies have used an appropriate comparison group^14–18^ and only two of them observed positive association with candidate genes, particularly with genes involved in DNA repair and apoptosis (*TP53*) and in DNA synthesis/repair (*MTHFR*)^17,18^. Ding *et al*.^18^ observed synergistic impact of single nucleotide polymorphisms (SNPs) in *TP53* and *MTHFR* on t-MN after Hodgkin lymphoma; however, Guillem *et al*.^17^ reported that *MTHFR* risk haplotype is variable depending on the primary neoplasia. Therefore, it seems that *TP53* polymorphic variants might be related to a higher risk of t-MN. These studies need further replication and extension with functional approaches^13^.

The p53 protein, when upregulated after genotoxic stress caused by anticancer therapy, for example, induces pathways that ultimately lead to either cell cycle arrest and DNA damage repair or apoptosis. Interestingly, the *TP53* gene harbors a common SNP that results in expression of either arginine (Arg) or proline (Pro) at codon 72, the Arg72Pro polymorphism. There is experimental evidence in cell models that the Arg variant is significantly more efficient at inducing apoptosis, whereas the Pro variant is a more potent inductor of cell cycle arrest and DNA repair^19–21^. Ding *et al*.^18^ observed a higher risk for t-MN after lymphoma among Pro carriers of the Arg72Pro polymorphism.

Notably, Ellis *et al*.^22^ observed that individuals carrying both the *TP53* Pro allele and the *MDM2* G allele are at increased risk of t-AML, although neither polymorphism alone was associated with t-AML. The MDM2 protein is an important negative regulator of p53. MDM2 has been shown to inhibit p53 by regulating its nuclear export, its capacity as a transcriptional activator and its stability (reviewed in^23^). It has been observed that a SNP located in the core promoter of *MDM2* (SNP309, T/G) affects binding of the transcription factor SP1. Particularly, the G-allele is bound more efficiently by SP1 than the T-allele, resulting in high levels of MDM2 mRNA and protein, and subsequent attenuation of the p53 response^24^.

In the present study, we wanted to investigate whether polymorphisms in the p53 pathway can be useful for predicting t-MN susceptibility. First, we aimed at determining if the Pro variant of Arg72Pro polymorphism at *TP53* and the G allele at *MDM2* SNP309 have an influence on the t-MN risk comparing the genotypes of a series of patients with this disease versus an appropriate control group. Moreover, we wanted to compare in a cell-based model, after treatment with an alkylating agent (busulfan) or a topoisomerase II poison (doxorubicin), the differences between the two *TP53* polymorphic variants concerning i) the number of DNA DSBs, measured by counting phosphorylated H2AX (γH2AX) foci^25^, ii) levels of DNA damage, measured as chromosome breaks or sister chromatid exchanges (SCEs), iii) the number of apoptotic cells, and finally iv) the development of chromosomal abnormalities typical of t-MN after a long term culture.

## Results

### SNP genotyping of *TP53* and *MDM2*

Forty and 39 t-MN samples were successfully genotyped for the *TP53* Arg72Pro polymorphism and the *MDM2* SNP309, respectively. Regarding controls, 54 samples were successfully genotyped for the *TP53* Arg72Pro polymorphism and 47 for the *MDM2* SNP309. There were 34 t-MN samples and 35 controls with genotype information for both *TP53* and *MDM2* genes. Unfortunately, not all samples could be genotyped for both genes due to lack of good-quality DNA. Supplementary Table S2 shows the genotype distribution of both SNPs. *TP53* followed the Hardy-Weinberg equilibrium, but not *MDM2* (p = 0.04). The Pro variant of the *TP53* Arg72Pro polymorphism was more frequent in the patients with t-MN; however, the association was not statistically significant, probably due to the small population size (log-additive model: OR = 1.69, 95% confidence interval [CI] = 0.90–3.17, p = 0.096; recessive model, Pro/Pro vs. Arg/Arg + Pro/Arg: OR = 3, 95% CI = 0.70–12.82, p = 0.125). On the other hand, *MDM2* SNP309 was highly significantly associated with t-MN risk in log-additive model (OR = 2.49, 95% CI = 1.34–4.61, p = 0.002), in dominant model (T/G + G/G vs. T/T: OR = 3.75, 95% CI = 1.51–9.31, p = 0.003) and in codominant model (T/G vs. T/T: OR = 3.09, 95% CI = 1.14–8.35; G/G vs. T/T: OR = 5.60, 95% CI = 1.58–19.87; p = 0.009). These associations were maintained after adjustment by sex. The synergistic effect of both genes was tested and significant association was found between *TP53* and *MDM2* genotypes and risk of t-MN. The *TP53* Arg/Arg and *MDM2* T/T genotype was underrepresented in t-MN patients compared to non-t-MN individuals (6% vs. 29%, p = 0.013, chi-square test). Moreover, the only case with the *TP53* Pro/Pro and *MDM2* G/G genotype had a t-MDS. The *TP53* Arg/Arg or Pro/Arg and *MDM2* T/T genotype was less frequent in t-MN patients compared to non-t-MN individuals (18% vs. 40%, p = 0.041, chi-square test), whereas the *TP53* Pro/Pro and *MDM2* G/G or T/G genotype was more frequent in t-MN patients, even though differences were not statistically significant (9% vs. 0%, p = 0.114, Fisher exact test).

### Analysis of γ-H2AX foci

**Figure 1:**
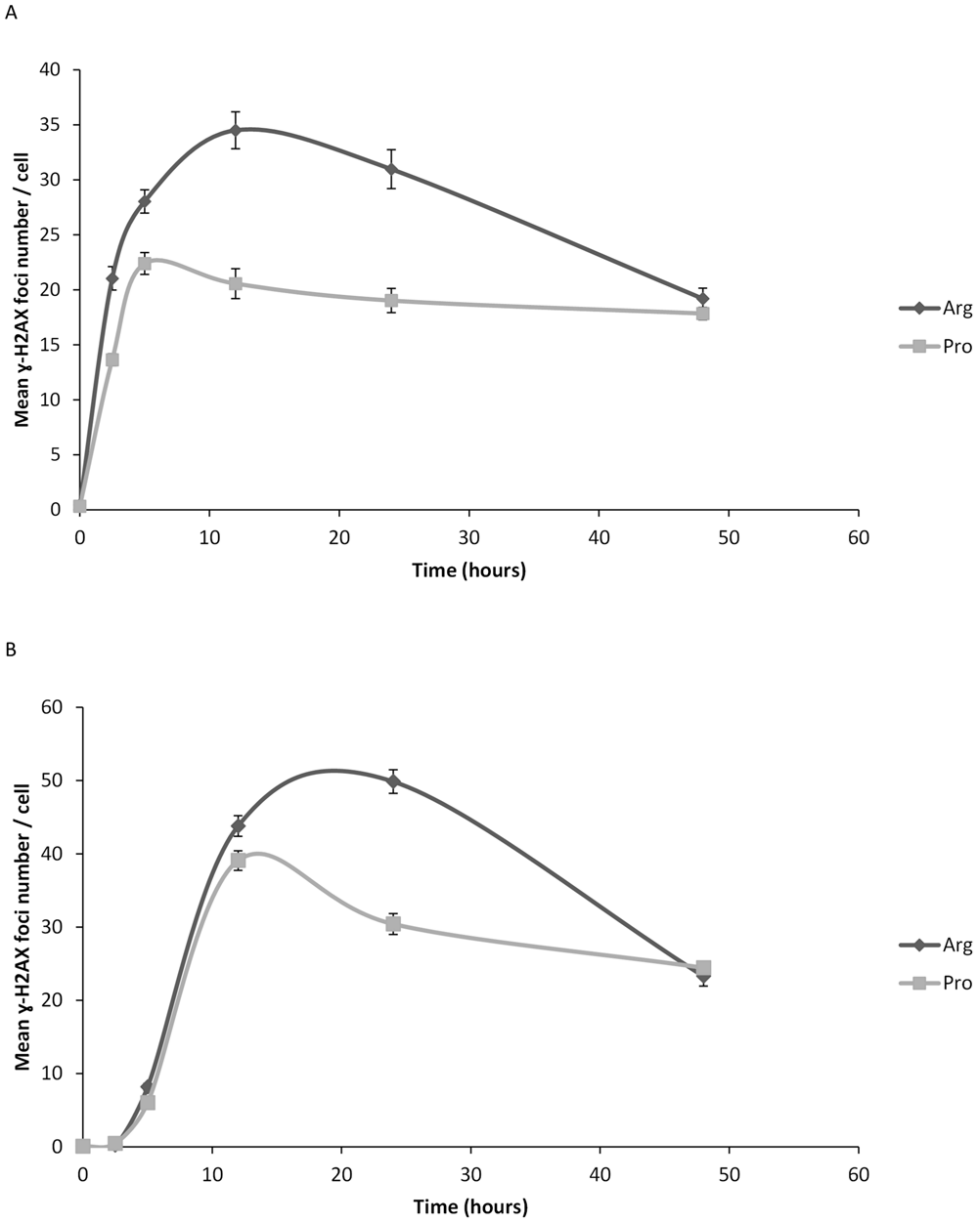
Kinetics of γ-H2AX foci formation and disappearance in Jurkat p53Arg and Jurkat p53Pro cells after treatment with busulfan (**A**) or doxorubicin (**B**) for 2 h. Values correspond to means ± SEM and results are representative of two independent experiments.

We next addressed *in vitro* whether the two polymorphic variants of *TP53* result in distinct cellular responses to anticancer drugs in isogenic Jurkat cell lines expressing p53Arg and p53Pro. First, γ-H2AX foci kinetics was assessed after drug treatment (Fig. 1A,B, Supplementary Table S3 and Fig. S1). We observed foci (>1 foci/cell) after 2.5 h in cells treated with busulfan and after 5 h in cells treated with doxorubicin. After foci were detectable, Jurkat p53Arg cells presented a significantly higher number of foci per cell than Jurkat p53Pro cells at all times tested and for both treatments, except at 48 h (p values are shown in Supplementary Table S3). Maximum values of γ-H2AX foci were detected at 15–20 h post-treatment for Jurkat p53Arg, and at 5–15 h for Jurkat p53Pro.

### Chromosome breakage assay and SCE assay

**Figure 2:**
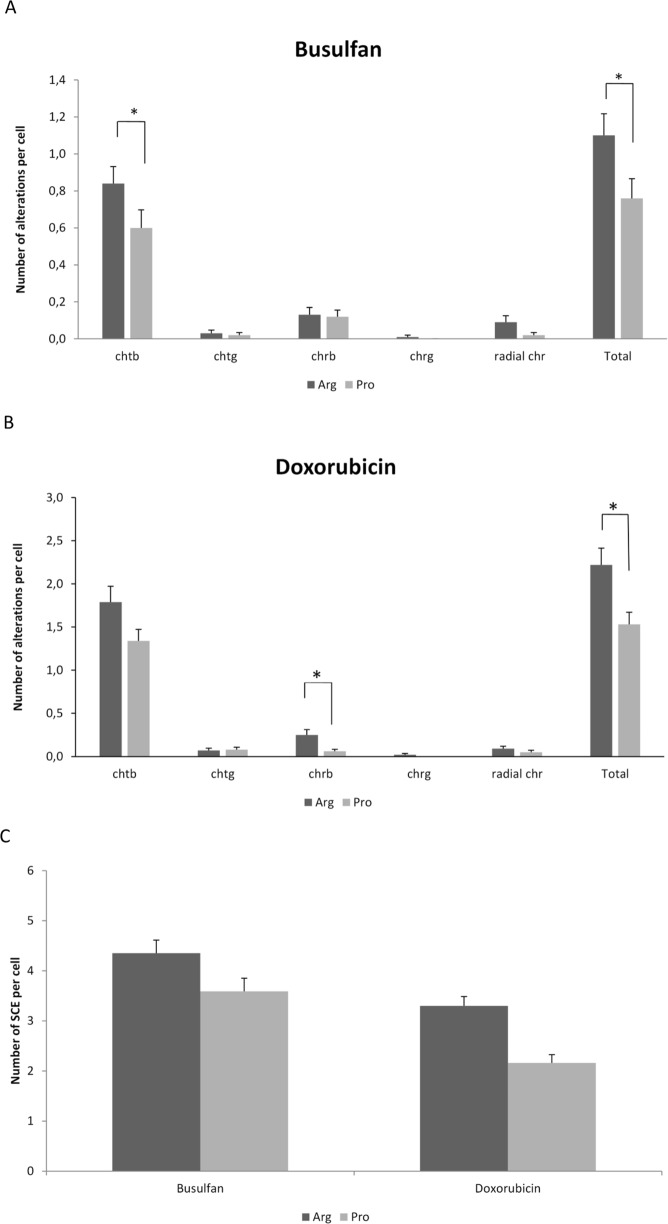
DNA damage in Jurkat p53Arg and Jurkat p53Pro cells treated with busulfan or doxorubicin. (**A**,**B**) Chromosome alterations per cell (chtb, chtg, chrb, chrg, radial chromosomes) in Jurkat p53Arg and p53Pro treated with busulfan (**A**) or doxorubicin (**B**) for 2 h and cultured for 24 h; (**C**) Number of SCE per cell after treatment with busulfan or doxorubicin for 2 h and culture of 48 h. Data are plotted as means ± SEM and results are representative of two independent experiments. Asterisks denote significant differences (*p < 0.05; ***p < 0.001, Mann-Whitney test).

We evaluated the effect of the *TP53* polymorphism on the number of breaks on chromosomes (chrb) or chromatids (chtb) in the p53Pro- and p53Arg-expressing isogenic cell lines 24 h after drug treatment (Fig. S2). Jurkat p53Arg cells had a statistically significantly higher number of chtb (p = 0.016) and total chromosomal aberrations (p = 0.018) than Jurkat p53Pro cells after treatment with busulfan (Fig. 2A). On the other hand, Jurkat p53Arg cells treated with doxorubicin had more chtb than Jurkat p53Pro cells but differences were not statistically significant (p = 0.184); moreover, they had a statistically significantly higher number of chrb (p = 0.012) and total chromosomal aberrations (p = 0.021) than Jurkat p53Pro cells (Fig. 2B).

To further investigate the difference in DNA damage induction, we also analyzed the levels of SCE 48 h after drug treatment using the same cells (Fig. S3). Consistent with the data from chromosome breakage assay at 24 h, SCE assay demonstrated that Jurkat p53Arg cells had more SCE per cell than Jurkat p53Pro cells 48 h after treatment with busulfan (p = 0.02) or doxorubicin (p = 5 × 10^−6^) (Fig. 2C).

### Cell proliferation

**Figure 3:**
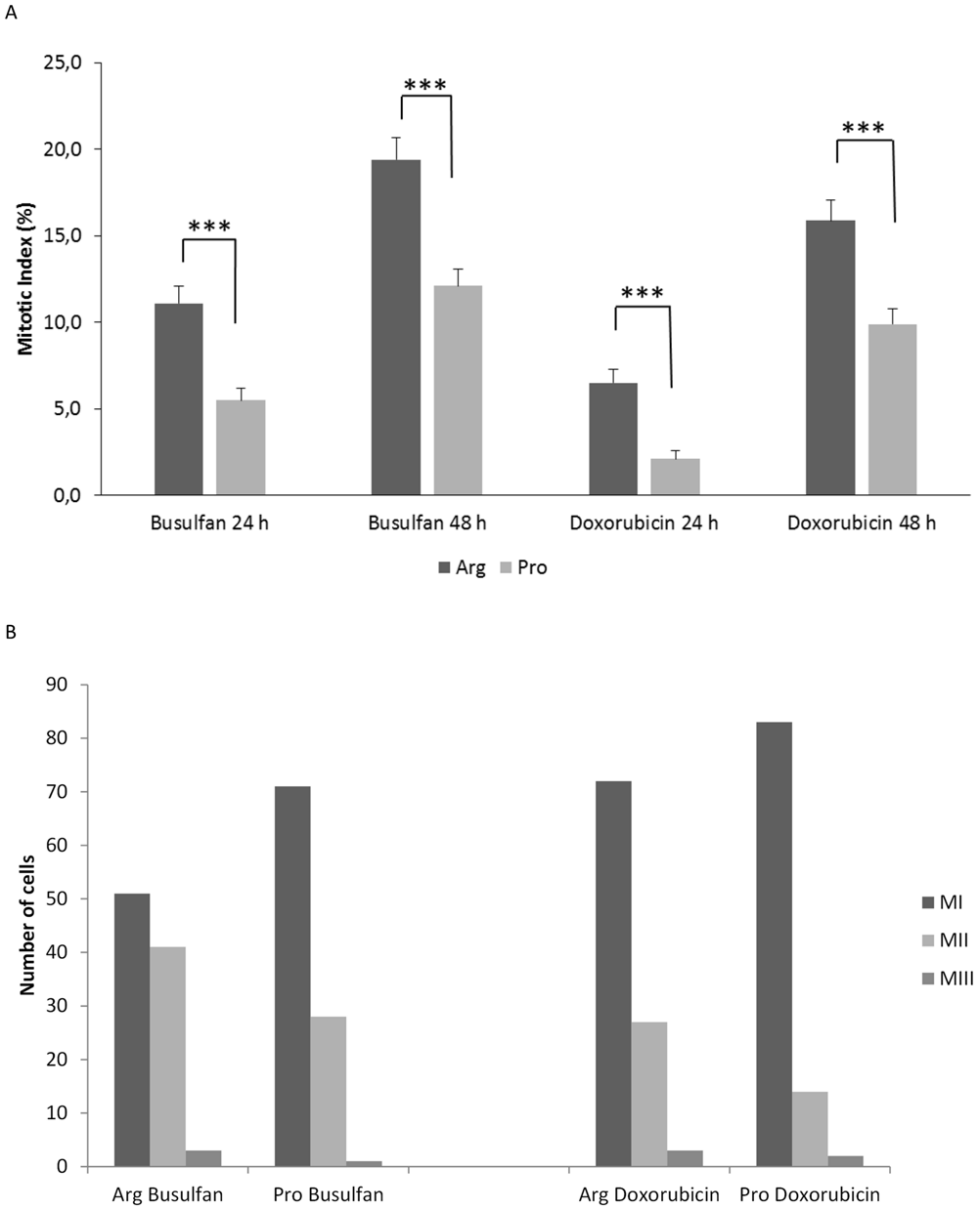
Cell proliferation in Jurkat p53Arg and Jurkat p53Pro cells treated with busulfan or doxorubicin for 2 h. (**A**) Mitotic index in Jurkat p53Arg and Jurkat p53Pro cells treated with busulfan or doxorubicin for 2 h and cultured for 24 h or 48 h; (**B**) Number of cells at MI, MII, or MIII in Jurkat p53Arg and Jurkat p53Pro cells treated with busulfan or doxorubicin for 2 h and cultured for 48 h. Results are representative of two independent experiments. Asterisks denote significant differences (***p < 0.001, z-test).

We next evaluated whether mitotic and proliferation indexes would also be affected by p53Arg or p53Pro expression. Untreated Jurkat p53Arg cells and Jurkat p53Pro cells had a mitotic index of 28.8% and 27%, respectively, at 24 h, and 38.1% and 39.6%, respectively, at 48 h. These differences were not statistically significant. However, the cells presented differences in their mitotic index after treatment with drugs (Fig. 3A). Jurkat p53Arg treated with either busulfan or doxorubicin divided more actively than Jurkat p53Pro both at the 24-h and 48-h cultures (p < 0.001 in all cases).

The proliferation index for doxorubicin-treated cells was 1.32 ± 0.12 in Jurkat p53Arg cells and 1.18 ± 0.23 in Jurkat p53Pro cells, whereas for busulfan-treated cells, it was 1.49 ± 0.15 in Jurkat p53Arg cells and 1.30 ± 0,09 in Jurkat p53Pro cells. As can be observed in Fig. 3B, Jurkat p53Pro cells presented more cells in mitosis I (MI) and fewer cells in MII than p53Arg cells after chemical treatment, suggesting that more cells were arrested at MI.

### Apoptosis assay

**Figure 4:**
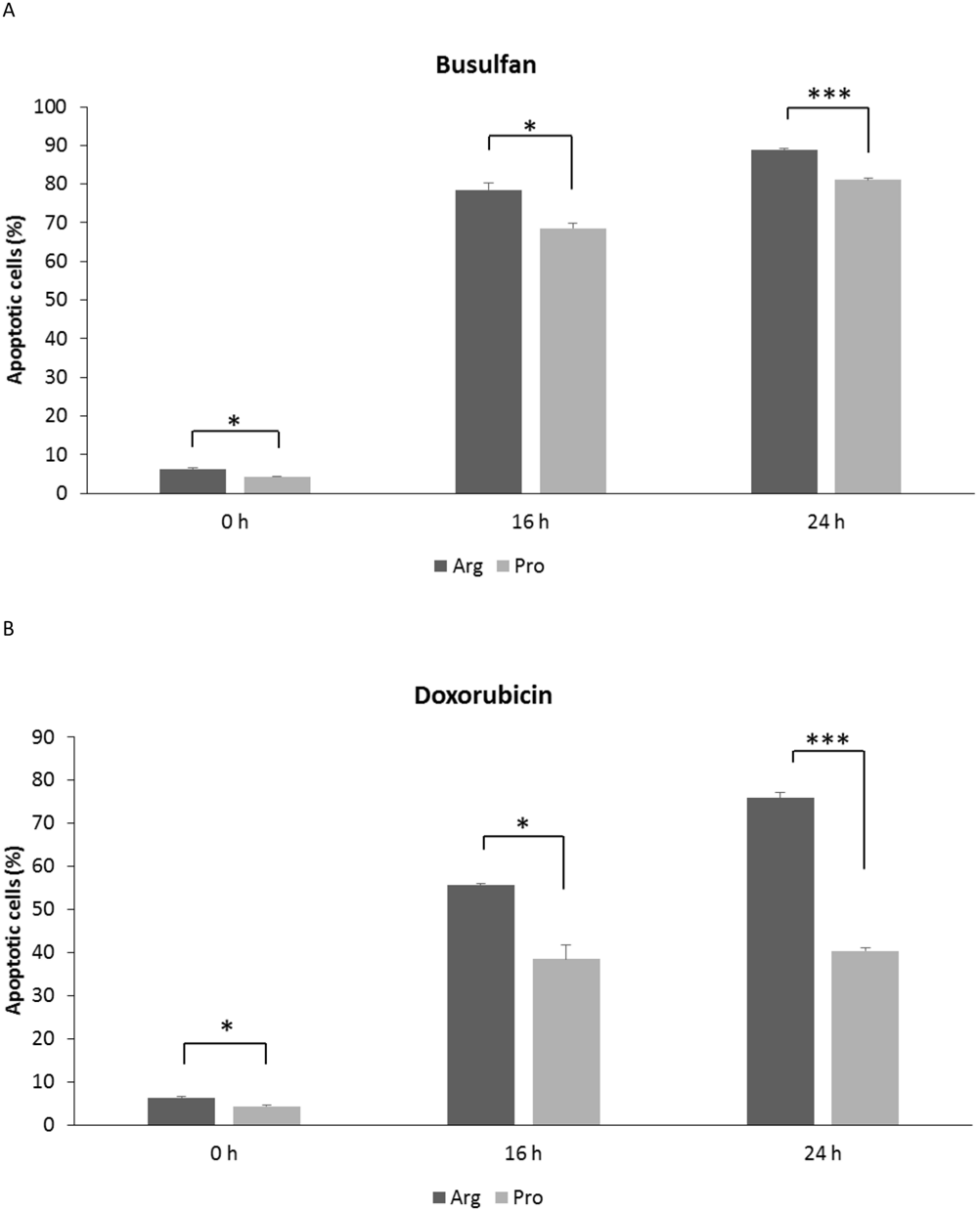
Apoptosis potential of Jurkat p53Arg and Jurkat p53Pro cells after treatment with busulfan (**A**) or doxorubicin (**B**) for 2 h. Data are plotted as mean ± SEM of three independent experiments. Asterisks denote significant differences (*p < 0.05; ***p < 0.001, t-test).

We next determined whether there were any differences in the apoptosis-induction potential between the isogenic cells expressing p53Arg or p53Pro (Fig. S4). The results revealed that Jurkat p53Arg cells had a consistently higher level of apoptosis than Jurkat p53Pro cells after treatment with either busulfan or doxorubicin at all points analyzed, and even with no drug treatment, which corresponds to spontaneous cell death (Fig. 4).

### FISH analysis and PCR

**Figure 5:**
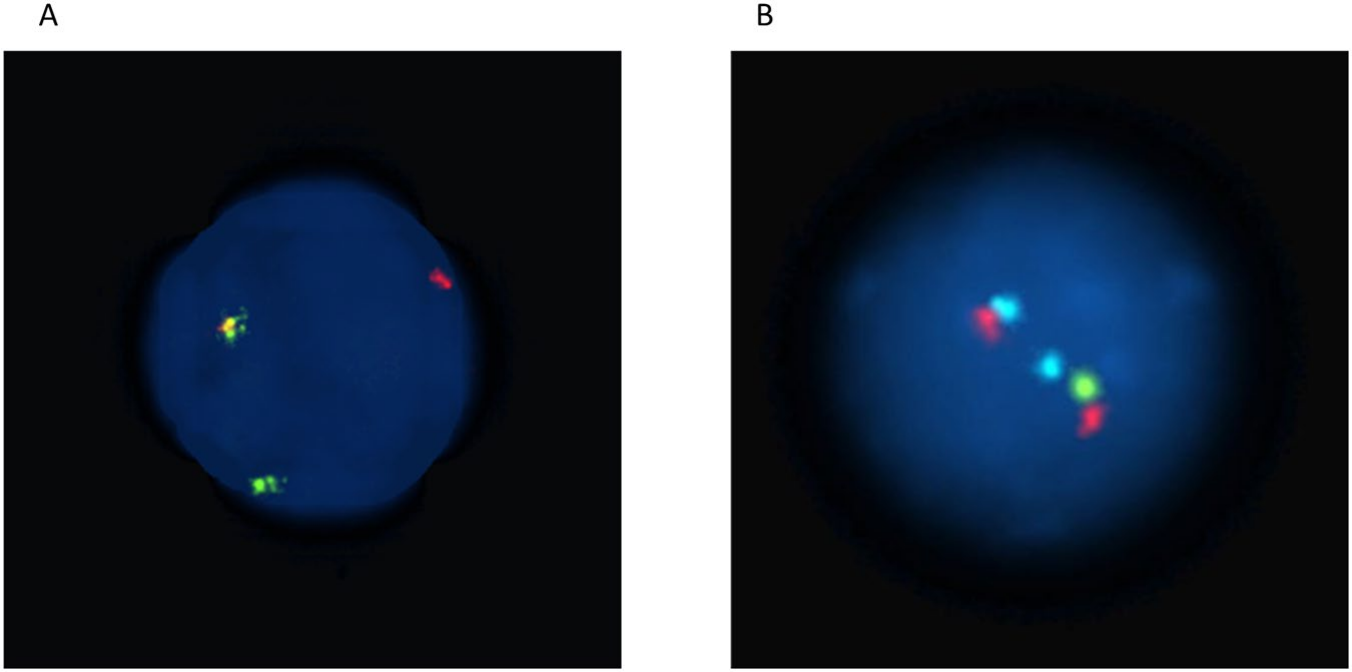
FISH images of nuclei from the Jurkat p53Pro cell line after a 100-day culture using the translocation *PML*-*RARA* specific probe (**A**) or the del(5q) specific probe (**B**). (**A**) Green signal corresponds to *RARA* gene on chromosome 17, red signal corresponds to *PML* gene on chromosome 15, fusion signal corresponds to translocation t(15;17). (**B**) Blue signal corresponds to 5p15, red signal corresponds 5q31, and green signal corresponds to 5q33. This cell has a deletion of 5q33.

Then, a FISH analysis and PCR was carried out to evaluate if Jurkat p53Pro cells presented some of the chromosomal/molecular abnormalities typical of t-AML after a long-term culture of 100 days. The analysis showed that either Jurkat p53Arg or Jurkat p53Pro cells did not present the *MLL* rearrangement after treatment with doxorubicin. Interestingly, Jurkat p53Pro cells had 6.3% and 15.5% of nuclei with the t(15;17) *PML-RARA* translocation after treatment with doxorubicin in the two experiments performed (Fig. 5A), whereas Jurkat p53Arg cells did not present the rearrangement. Many hiperdiploid cells were observed in the doxorubicin-treated cultures: 52% of metaphases in Jurkat p53Pro cells and 51% in Jurkat p53Arg cells, as expected given the implication of topoisomerase II enzyme in sister chromatid separation (reviewed in^26^). On the other hand, either Jurkat p53Arg or Jurkat p53Pro cells did not present deletion of chromosome 7 after treatment with busulfan, but Jurkat p53Pro cells had deletion at chromosome 5. Two percent of cells and 26.9% of cells presented deletion of 5q33 after treatment with busulfan in the two experiments performed (Fig. 5B), whereas Jurkat p53Arg cells did not present the deletion. None of the cell lines presented *FLT3*-ITD or *NPM1* mutations.

## Discussion

One of the most severe complications after successful cancer therapy is the development of a secondary cancer, mostly an acute leukemia of myeloid lineage (t-MN). Unfortunately, the number of these therapy-related cancers has constantly increased over recent decades, because cancer patients are now surviving longer. Constitutional genetic variation is likely to impact on an individual’s risk of developing such a secondary cancer. Therefore, the analysis of genetic polymorphisms may help to identify patients at high risk of developing t-MN, which might benefit from surveillance and/or from personalized treatment.

In the present study, we investigated the impact of polymorphisms in the p53 pathway on the development of t-MN in a series of 45 t-MN patients and their appropriate controls. We observed that the *TP53* Arg72Pro polymorphism and the *MDM2* SNP309 were associated with t-MN risk. The Pro variant of *TP53* and the G allele of *MDM2* were overrepresented in t-MN patients as compared to non-t-MN individuals. This confirms previous results by^22^ for *TP53* and *MDM2* and by^18^ for *TP53*. It is well known that the two forms of p53 at codon 72 have differences in their abilities to induce apoptosis and suppress transformed cell growth^19^. As regards to *MDM2* SNP309, the homozygous G/G or heterozygous T/G genotypes have been shown to be associated with significantly higher levels of MDM2 expression in normal and tumoral cells, accompanied by suppression of p53 and attenuation of the p53 apoptotic response after exposure to chemotherapy agents (reviewed in^23^). Moreover, the G allele has been linked to increased cancer risk for many human tumors and in mouse models; however, some other reports have failed to obtain the same results (reviewed in^27^).

Overall, the above-mentioned reports and the association study presented here suggest the p53 pathway as a likely t-MN risk factor. To analyze this further, we developed a cellular model with isogenic Jurkat cell lines expressing the Arg and the Pro variant of the *TP53* Arg72Pro polymorphism, in order to see whether the altered biochemical and/or biological function of p53 due to polymorphisms can have an effect on cells after treatment with chemotherapy agents.

A summary of the results obtained in the cell-based model is shown in Table 1. Regarding γ-H2AX foci, Jurkat p53Arg cells presented more foci per cell than Jurkat p53Pro cells after treatment with busulfan or doxorubicin. Previous studies have reported that γ-H2AX foci analysis can reflect DSB repair capacity: repair-deficient mouse strains and radiosensitive cells/mice present increased foci levels compared to repair-proficient mouse strains and radioresistant cells/mice, respectively^28–30^. This means that if Jurkat p53Pro cells had fewer DSBs, it is probably due to the fact that expression of the Pro variant results in a significantly higher DNA-repair capacity than the Arg variant, as others have observed^21^. The Pro variant activates transcription of several p53-dependent target genes involved in DNA repair (i.e. *GADD45*, *DDB2*, and *RRM2B*) more efficiently than the Arg form^21^. Moreover, Siddique & Sabapathy^21^ evaluated the correct NHEJ activity, which rejoins broken DNA ends, and observed that the Pro form is more potent than the Arg form in the repair of exogenously damaged plasmids, both in tumoral and non-tumoral mouse cells.

**Table 1:** Summary of results obtained in Jurkat p53Arg and p53Pro after drug treatment.

Parameter measured	Jurkat p53Arg	Jurkat p53Pro
Number of foci up to 24 h	↑	↓
Number of foci at 48 h	=	=
Number of chromosomal aberrations at 24 h	↑	↓
Number of SCE at 48 h	↑	↓
Mitotic index at 24 h and 48 h	↑	↓
Proliferation index at 48 h	↑	↓
Cell cycle arrest	↓	↑
Number of apoptotic cells at 16 h and 24 h	↑	↓
Chromosomal abnormalities at 100-day culture	Absent	Present

Unlike ionizing radiation, most DNA damaging drugs, such as alkylating agents and topoisomerase II poisons, do not induce DSBs directly. This explains why maximum values of γ-H2AX foci were observed several hours after treatment, as other authors have described^31^. Very late counts of as many as 20 γ-H2AX foci can correspond to cells with unrepaired DSBs or to cells that are likely to die^32^.

Regarding chromosome and chromatid breaks and SCE per cell, again Jurkat p53Arg cells presented a higher level of DNA damage than Jurkat p53Pro cells after drug treatment. Siddique & Sabapathy^21^ reported that Pro-expressing cells exhibit reduced micronuclei formation compared to Arg-expressing cells. Later, Litviakov^33^ showed that cancer patients with Pro/Pro genotype have fewer chromatid breaks in comparison to Arg/Pro and Arg/Arg carriers. Moreover, chromosomal radiosensitivity, as measured by the G2-chromosome break assay, has been reported to be associated with polymorphisms in *TP53* codon 72^34^. In that study, reduced frequencies of radiation-induced chromatid aberrations were observed in normal human lymphoblast cell lines with the Pro/Pro genotype compared to other genotypes.

We observed that chtb was the predominant type of chromosomal aberration, as would be expected for the mechanism of action of the two drugs used. Busulfan generates adducts, which cause replication stall during cell division, generating DSBs in the replication fork. If not properly repaired, these DSBs result in chtb in mitosis. On the other hand, doxorubicin inhibits the ligase activity of topoisomerase II, generating DSBs mostly behind the replication fork and near the transcription bubble^26^. When DSBs are generated behind the replication fork or during transcription in G2, and if they are not repaired, they result in chtb in mitosis. When generated during transcription in G1, and if breaks in both strands remain unrepaired through S phase, the result will be visible in both chromatids as a chrb in next mitosis^35^.

In relation to cell proliferation, we observed that Jurkat p53Arg cells treated with drugs for 2 h and cultured for 24 h and 48 h divided more actively (higher mitotic and proliferation indexes and more MII cells) than Jurkat p53Pro cells, even though the mitotic index was low (maximum values were 55% of the untreated cells index). On the other hand, more Jurkat p53Pro cells were arrested at MI. It is known that expression of the Pro variant in cell lines results in a predominant cell cycle arrest in G1^36^, most likely to allow cells to carry out DNA repair processes.

As for apoptosis, Jurkat p53Arg cells presented higher apoptotic potential than p53Pro cells after treatment with busulfan or doxorubicin for 16 h or 24 h. Back in 1999, Thomas *et al*.^19^ reported that the two forms of p53 have differences in their transcriptional activities and in their abilities to induce apoptosis and suppress transformed growth. Several reports published later confirmed that the Arg variant induces apoptosis more efficiently than does the Pro variant, probably due to its enhanced localization to the mitochondria^37^, to its more efficient induction of specific apoptosis-associated genes, e.g. *NOXA*, *PUMA*, *PIGPC1* and *AIP1*^36,38^, or even to its ability to escape from negative regulation by iASPP^39^. Most of these studies were performed on tumoral cells but Jeong *et al*.^38^ included also non-tumoral cells with identical results.

Finally, we were able to detect the t(15;17) translocation and del(5)(q33) in Jurkat p53Pro cells, but not in Jurkat p53Arg cells, after treatment with doxorubicin or busulfan, respectively, and after a long-term culture. These chromosomal abnormalities are likely to be related to DNA breaks that have been repaired aberrantly^40^. Other authors have reported the t(15;17) translocation in patients with t-AML, especially arising after treatment with anthracyclines (doxorubicin, epirubicin, or daunorubicin) and/or mitoxantrone^41,42^. Moreover, happloinsufficiency of the *RPS14* gene on 5q33 has been implicated in the pathogenesis of MN, even though mutations in other genes are needed for the pathogenesis and progression of t-MN^43^.

**Figure 6:**
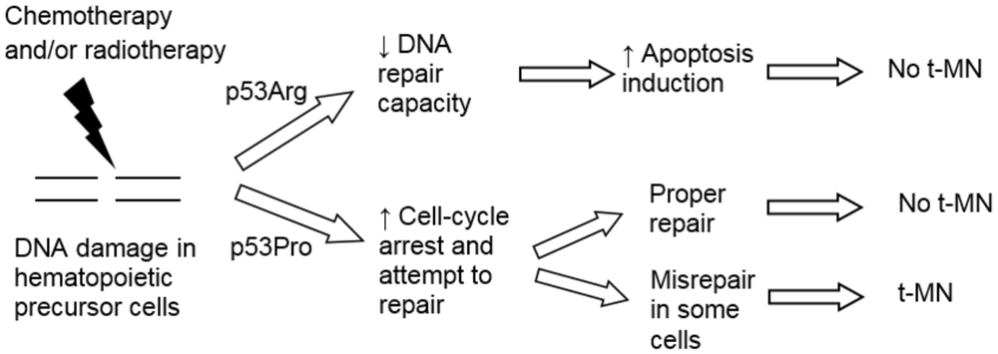
Hypothetical model of t-MN development after treatment with chemotherapy and/or radiotherapy. The capacity of cells to undergo apoptosis or to arrest cell cycle and repair DNA damage more or less efficiently can be influenced by genetic polymorphisms in the p53 pathway, for example, the *TP53* Arg72Pro polymorphism.

Taken together, the results from the present study showed that the p53Arg-expressing cells presented an increased rate of DNA damage compared to p53Pro-expressing cells after treatment with the same dose of busulfan or doxorubicin. These lesions were probably incompletely or inefficiently repaired and the apoptotic program was triggered. As most cells with high levels of DNA damage died, no chromosomal abnormalities were observed in Jurkat p53Arg cells. On the other hand, p53Pro-expressing cells had fewer DNA breaks, probably due to cell cycle arrest and DNA damage repair. Therefore, these cells were less prone to perform apoptosis. In some of these Jurkat p53Pro cells, DNA repair was erroneous, generating chromosomal abnormalities typical of t-MN observed after a long-term culture. Figure 6 shows a model of possible mechanisms underlying differences in t-MN risk in patients treated with the same doses of chemotherapy and/or radiation according to the ability of individuals to undergo apoptosis or to repair DNA lesions more or less efficiently related to the *TP53* polymorphism.

In conclusion, the association study presented here showed that the Pro variant of Arg72Pro polymorphism at *TP53* and the G allele at *MDM2* SNP309 had an influence on the t-MN risk. Moreover, we observed in isogenic cell lines that the two *TP53* polymorphic variants at codon 72 were functionally distinct; they had different effects on DNA damage levels and on generation of chromosomal abnormalities after treatment with an alkylating agent or a topoisomerase II poison. We suggest that these differences may influence t-MN risk. Previous studies have also reported that p53Arg is more efficient in suppressing malignant transformation than p53Pro^19,38^. Our association analysis indicated that there must be other genes, probably from the p53 pathway, that can be modulating the t-MN risk, e.g. *MDM2* SNP309. Other p53 pathway proteins that interact with or are under the control of p53 have been found to contain polymorphisms of potential clinical interest, such as ATM and p21, among others (reviewed in^44^). It will be very important to confirm the effect of p53-pathway polymorphisms on t-MN risk in larger series of t-MN patients and their appropriate controls in a near future.

## Patients and Methods

### Patients

The study comprised 45 patients with t-AML or t-MDS, and a control group of 66 patients (37 males and 29 females) that had been diagnosed with acute leukemia, treated with chemotherapy (and in some cases with concomitant radiotherapy) and who 5 years or more after treatment had not developed a t-MN. All patients were informed about the study and provided their consent. The research was conducted in accordance with the Declaration of Helsinki, and it was approved by the Clinical Research Ethics Committee of Vall d’Hebron Hospital from Barcelona, Spain and by the Clinical Research Ethics Committee of Jose María Morales Meseguer Hospital from Murcia, Spain. Clinico-biological characteristics of t-MN patients are shown in Supplementary Table S1.

### DNA isolation and genotyping

DNA was extracted following standard methods described in Supplementary Methods. The samples were genotyped for the *TP53* Arg72Pro SNP and the *MDM2* SNP309 by the Polymerase Chain Reaction - Restriction Fragment Length Polymorphism (PCR-RFLP) method, following the procedure proposed by^45^ and^46^, respectively (Supplementary Methods).

### Cell lines

To directly assess the biological effect of the *TP53* polymorphism on cells treated with chemotherapy agents, we established isogenic cell lines expressing p53Arg or p53Pro. Unfortunately, *MDM2* null cells are not viable and such a model could not be constructed for the *MDM2* gene. Details about cell culture, plasmids, retroviral production and infection are described in Supplementary Methods.

### Immunofluorescence staining and analysis of γ-H2AX foci

To assess the kinetics of γ-H2AX foci induction and disappearance following drug treatment, immunostaining of foci and microscopic analysis was performed as described in Supplementary Methods.

### Chromosome breakage assay, SCE assay, and cell proliferation

After drug treatment, we performed chromosome breakage assay and SCE assay as described in Supplementary Methods. Cell proliferation was also measured.

### Apoptosis assay

Apoptosis was assessed after drug treatment, following manufacturer’s instructions (Supplementary Methods).

### Fluorescence *in situ* hybridization (FISH) and PCR

Cell cultures treated with drugs were grown for 100 days. FISH assay was performed to detect *MLL* rearrangement, t(15;17) *PML*-*RARA* and deletion of chromosome 5 or 7. Moreover, DNA was extracted and cells were genotyped for *FLT3*-ITD and *NPM1* mutations by PCR. Details are described in Supplementary Methods.

### Statistical analysis

Statistical analysis was performed as described in Supplementary Methods.

## Supplementary information

Supplemental Data

Supplemental Data

## Data Availability

All data generated or analysed during this study are included in this published article (and its Supplementary Information files).
