# Supplementary material for: Polymorphisms in MDM2 and TP53 Genes and Risk of Developing Therapy-Related Myeloid Neoplasms
Source: Sci Rep. 2019 Jan 17;9:150. doi: 10.1038/s41598-018-36931-x (PMC6336808; doi:10.1038/s41598-018-36931-x)
Supplement: Supplementary file 1 — Supplemental Data [file 41598_2018_36931_MOESM1_ESM.docx]

**POLYMORPHISMS IN *MDM2* AND *TP53* GENES AND RISK OF DEVELOPING THERAPY-RELATED MYELOID NEOPLASMS**

**Maria Cabezas^1^, Lydia García-Quevedo^1^, Cintia Alonso^1^, Marta Manubens^1^, Yolanda Álvarez^1^, Joan Francesc Barquinero^1^, Santiago Ramón y Cajal^2, 3^, Margarita Ortega^4^, Adoración Blanco^4^, María Rosa Caballín^1^, Gemma Armengol^1*^**

^1^ Unit of Biological Anthropology, Department of Animal Biology, Plant Biology and Ecology, Faculty of Biosciences, Universitat Autònoma de Barcelona, 08193-Bellaterra, Catalonia, Spain

^2^ Department of Pathology, Vall d’Hebron University Hospital, 08035-Barcelona, Catalonia, Spain

^3^ Spanish Biomedical Research Network Centre in Oncology (CIBERONC), Spain

^4^ Department of Hematology, Vall d’Hebron University Hospital, 08035-Barcelona, Catalonia, Spain

**SUPPLEMENTARY METHODS**

*Patients*

The study comprised 45 patients with t-AML or t-MDS, whose samples were obtained from the Laboratory of Clinical Analysis, Balagué Center, Barcelona, which received samples from all over Spain. Clinico-biological characteristics of t-MN patients are shown in Table S1. The study also included a control group of 66 patients (37 males and 29 females) that had been diagnosed with acute leukemia, treated with chemotherapy (and in some cases with concomitant radiotherapy) and who 5 years or more after treatment had not developed a t-MN (mean=103 months, SD=33 months, range=60-199 months). These patients had received various chemotherapy agents, including both alkylating agents and topoisomerase II poisons. All participants in the study were of Caucasian descent. None of the patients had undergone bone marrow transplant.

*DNA isolation and genotyping*

DNA was extracted from bone marrow cell nuclei in fixative (acetic acid-methanol, 1:3) using the standard phenol-chloroform method or the QIAamp DNA Mini Kit (Qiagen, Hilden, Germany) following manufacturer’s instructions. The first steps were dilution of the cell pellet to an OD600 of approx. 1.0 in fixative and removal of the fixative with two PBS washes.

The samples were genotyped for the *TP53* Arg72Pro SNP and the *MDM2* SNP309 by the Polymerase Chain Reaction - Restriction Fragment Length Polymorphism (PCR-RFLP) method, following the procedure proposed by ^1,2^, respectively, with the following modifications. PCR was performed in a 20-μL (*TP53*) or 25-μL (*MDM2*) reaction volume containing 100 ng of genomic DNA, 1x PCR-buffer, 0.2 μM of each primer, 200 μM dNTPs, 1.5 mM MgCl_2_, and 0.5 U Taq polymerase (Promega Corporation, Madison, WI). Cycling conditions were: initial denaturation at 94°C for 5 (*TP53*) or 1 min (*MDM2*) followed by 35 (*TP53*) or 30 (*MDM2*) cycles of denaturation at 94°C for 30 s, annealing at 58°C for 20 s (*TP53*) or 30 s (*MDM2*), and extension at 72°C for 20 s (*TP53*) or 30 s (*MDM2*), and a final extension step at 72°C for 2 min (*TP53*) or 10 min (*MDM2*). The PCR product (10 μL) was digested with BstUI (*TP53*) or MspA1 I (*MDM2*) restriction enzymes (10 U/μL; Thermofisher Scientific, Rockford, IL) according to manufacturer’s specifications; digestion products were subjected to electrophoresis on a 3% agarose gel. In the case of *TP53*, the Arg homozygous genotype (G/G) was cleaved by BstUI and yielded two small fragments (160 and 119 bp), whereas the Pro homozygous genotype (C/C) was not cleaved and yielded a single 279 bp band. The heterozygote showed three bands (279, 160, and 119 bp). Regarding *MDM2* digestion, the wild-type homozygous genotype (T/T) yielded one band (233 bp), the heterozygous genotype (G/T) yielded three bands (233, 187, and 46 bp) and the other genotype (G/G) yielded two bands (187 and 46 bp).

*Cell culture and plasmids*

To directly assess the biological effect of the *TP53* polymorphism on cells treated with chemotherapy agents, we established isogenic cell lines expressing p53Arg or p53Pro. Jurkat cells, which are *TP53* null and which are derived from a human lymphoblastic T-cell leukemia, were used (American Type Culture Collection, ATCC). Unfortunately, *MDM2* null cells are not viable and such a model could not be constructed for the *MDM2* gene. Jurkat cells were grown in RPMI-1640 medium containing 2 mM L-glutamine (Invitrogen, Carlsbad, CA) supplemented with 15% fetal bovine serum and 1% penicillin-streptomycin.

Plasmids expressing either p53Arg (pcDNA3.1-p53Arg) or p53Pro (pcDNA3.1-p53Pro) were kindly donated by Dr. Lawrence Banks and Dr. Miranda Thomas from the International Centre for Genetic Engineering and Biotechnology, Trieste, Italy ^3^. Inserts from these plasmids were subcloned into the pLNCX2 retroviral vector to construct pLNCX2-p53Arg and pLNCX2-p53Pro (HindIII and NotI sites) following standard methods.

*Cell transfection and retroviral transduction*

To generate cell lines that stably express p53Arg or p53Pro, retroviral production and infection was carried out. First, pLNCX2-p53Arg and pLNCX2-p53Pro constructs were transiently transfected into the Phoenix packaging cell line with jetPEI^®^ (Polyplus, Illkirch, France) according to the manufacturer's protocol. For retroviral infection, Jurkat cells were incubated in the presence of the retrovirus-containing supernatant and 4 µg/mL polybrene (Sigma-Aldrich, Taufkirchen, Germany) for 24 h. Infection was repeated the next day. Twenty-four hours after the second infection, medium supplemented with G418 (1 mg/mL, Sigma-Aldrich) was added, and cells underwent selection for 3 days to eliminate uninfected cells. Standard Western blot analysis was carried out to confirm p53 expression.

*Immunofluorescence staining and analysis of γ-H2AX foci*

To assess the kinetics of γ-H2AX foci induction and disappearance following drug treatment, immunostaining of foci and microscopic analysis was performed. Early log phase Jurkat p53Pro and Jurkat p53Arg cells were treated for 2 h with doxorubicin (500 nM) or busulfan (500 µM). After treatment, cell cultures were maintained at 37°C for 2.5, 5, 12, 24 and 48 h. Then, cells (30,000 cells/mL) were cytospun onto glass slides (Menzel-Glaser, Badalona, Spain) at 500 g for 5 min and immunofluorescence staining was performed as previously described ^4^. Automated slide scanning was done with a Zeiss Axio Imager.Z2 epifluorescence microscope (Metasystems, Altlussheim, Germany) and the MetaCyte software module of the Metafer4 Slide Scanning System v3.10.2 (Metasystems). The images were captured using a 63x PlanApo objective and an SpOr filter. All signals were acquired as a z-stack with 10 focal planes and a z-step size of 0.35 µm between planes. A unique classifier was used to count a minimum of 200 cells for each particular experimental condition (a previous experiment with this classifier showed that the number of foci scored in 100 cells is enough to obtain a satisfactory result ^4^). These experiments were performed two independent times.

*Chromosome breakage assay*

After treatment with doxorubicin (500 nM) or busulfan (500 µM) for 2 h, cells were incubated for 24 h in the presence of colcemid (0.15 μg/mL, Gibco Thermofisher Scientific, Barcelona, Spain). Then, cells were collected by centrifugation and hypotonic shock was induced by a 0.075 M potassium chloride solution. Cell suspension was fixed in a mixture of methanol and glacial acetic acid (v/v 3:1) and cells were dropped onto slides and air-dried before staining with Leishman stain (Leishman eosin methylene blue solution modified, Merck, Madrid, Spain). One hundred metaphases with 46 chromosomes were analyzed for each cell line and for each treatment in a Zeiss Axio Imager Z2 microscope coupled to a Metafer® Slide Scanning System v3.10.2. Chromosome gaps (chrg) and breaks (chrb), as well as chromatid gaps (chtg) and breaks (chtb) were classified according to the International System for Human Cytogenetic Nomenclature ^5^. The experiment was performed two independent times.

*SCE assay*

Cells were treated with doxorubicin (500 nM) or busulfan (500 µM) for 2 h and then incubated at 37°C for 48 h under the presence of the thymine analogue 5-bromo-2´-deoxyuridine (BrdU, 12 μg/mL, Sigma-Aldrich). Colcemid was added at a final concentration of 0.15 μg/mL 24 h before harvest. Harvesting, staining and microscope observation was performed as previously mentioned. However, to be able to observe SCE and chromosome breaks in the same metaphase, treatment to distinguish dark and pail chromatids was softer than standard methods for SCE analysis. Before Leishman staining, slides were treated with 0.15 mg/mL Hoechst 33258 (Sigma-Aldrich) for 15 min and then exposed to 254-nm Ultraviolet Crosslinker (UVP, Upland, CA) for an additional 60 min. Then, SCEs were scored in 100 MII cells (in second cell cycle) for each cell line and drug treatment. A SCE was counted each time that two adjacent segments of one of the chromatids in a chromosome were stained differently. These experiments were performed two independent times.

*Cell proliferation*

Cell proliferation was measured by the mitotic and proliferation indexes. The mitotic index was calculated as the ratio of the number of mitotic cells in 1000 stimulated nuclei, using the cultures from the chromosome breakage assay and the SCE assay. The proliferation index was determined as (MI+2MII+3MIII)/100 cells, using the cultures from the SCE assay. MI, MII, and MIII are the number of metaphase cells from the first, second and third cell cycle, respectively, according to the pattern of chromatid staining.

*Apoptosis assay*

To measure apoptosis after drug treatment, the Annexin-V-FLUOS Staining Kit (Roche, [Basel, Switzerland](https://www.google.es/search?biw=1366&bih=667&q=Basilea+Su%C3%AFssa&stick=H4sIAAAAAAAAAOPgE-LUz9U3MMqrzDZT4gAxDbNN0rS0spOt9POL0hPzMqsSSzLz81A4VhmpiSmFpYlFJalFxQCuCC6JQwAAAA&sa=X&ved=0ahUKEwjWodK5nI_PAhWBchoKHSW-D3sQmxMIwgEoATAU)) was used following manufacturer’s instructions. Cells were treated with 500 nM of doxorubicin or 500 µM of busulfan during 16 h and 24 h in a cellular incubator at 37°C. After labeling with Annexin-V-FLUOS, we determined the proportion of apoptotic cells using an Olympus BX-60 epifluorescence microscope (Olympus, [Shinjuku, Tokyo](https://www.google.es/search?espv=2&biw=1366&bih=623&q=Shinjuku&stick=H4sIAAAAAAAAAOPgE-LQz9U3SCssTlHiBLEMjSqyjLS0spOt9POL0hPzMqsSSzLz81A4VhmpiSmFpYlFJalFxQDEj7ymQwAAAA&sa=X&ved=0ahUKEwigvL_dsaXPAhUBFxQKHROUDRkQmxMInAEoATAS), Japan) equipped with FITC, Cy3 and DAPI filters, and a 50x Plan objective. Three independent experiments were performed analyzing 1000 cells for each particular experimental condition.

*Fluorescence in situ hybridization (FISH)*

Cell cultures treated with doxorubicin (500 nM) or busulfan (500 µM) for 2 h were grown for 100 days. Then, colcemid (0.15 μg/mL) was added 2 h before harvesting as previously mentioned. FISH assay was performed with the following probes for chromosomal alterations typical of t-MN: *MLL* dual color break apart rearrangement probe, t(15;17) *PML*-*RARA* dual color translocation probe, XL 5q31/5q33/5p15 locus-specific probe and a probe centromere-specific for chromosome 7 (CEP7). All the probes used were from Vysis, Downers Grove, IL, except chromosome 5 probe, which is from MetaSystems Probes, Heidelberg, Germany. Experiments were conducted two independent times and as previously described ^6^. The cut-off points for positive values were established as the mean of false positives in 1000 nuclei from bone marrow of five controls with no hematological malignancies plus three standard deviations. These cut-off values were 1.5 % for *MLL* rearrangements, 3.2% for t(15;17) *PML*-*RARA*, 1.1% for del(5)(q31), 1.2% for del(5)(q33), 1.5% for del(5)(q31-q33), and 9.8% for monosomy of chromosome 7.

*Polymerase chain reaction (PCR)*

Detection of *FLT3*-ITD and *NPM1* mutations was performed on cell cultures treated with doxorubicin or busulfan and grown in long-term culture; two independent experiments were performed. DNA was extracted following standard methods. The presence of *FLT3*-ITD was analyzed by PCR on genomic DNA as previously reported^7^. The type A mutation of the gene *NPM1* was studied with the commercial kit ipsogen® NPM1 mutA MutaQuant® (Qiagen) according to the manufacturer’s instructions.

*Statistical analysis*

Hardy-Weinberg equilibrium was explored for the two SNPs using a chi-square test. Test of association between *TP53* or *MDM2* polymorphisms and risk of developing t-MN was performed using the R 3.3.1 package (<http://www.r-project.org/>). Specifically, SNPassoc library ^8^, which implements logistic regression methods under five different genetic models (codominant, dominant, recessive, overdominant and log-additive), was used. Chi-square or Fisher exact test was used to assess association combining *TP53* and *MDM2* polymorphisms.

To compare the number of ɣH2AX foci, chromosome/chromatid breaks, SCE or the number of apoptotic cells between Jurkat p53Pro and Jurkat p53Arg cells after drug treatment, first, normality of distribution of these parameters was tested with a Kolmogorov-Smirnov test (with Lilliefors correction) or a Shapiro-Wilk test. In case of compliance with normal distribution, a parametric test (t-student) was used. Otherwise, the nonparametric Mann–Whitney test was applied. All these statistical analyses were carried out using SPSS v22.0 software. To compare the mitotic index between Jurkat p53Pro and Jurkat p53Arg cells after drug treatment, a z-test was applied with a z-score calculator for two population proportions (<http://www.socscistatistics.com/tests/ztest>).

REFERENCES

1. Li, F. *et al.* Association of p53 codon 72 polymorphism with risk of second primary malignancy in patients with squamous cell carcinoma of the head and neck. *Cancer* **116,** 2350–2359 (2010).

2. Walsh, C. S., Miller, C. W., Karlan, B. Y. & Koeffler, H. P. Association between a functional single nucleotide polymorphism in the MDM2 gene and sporadic endometrial cancer risk. *Gynecol. Oncol.* **104,** 660–664 (2007).

3. Thomas, M. *et al.* Two polymorphic variants of wild-type p53 differ biochemically and biologically. *Mol. Cell. Biol.* **19,** 1092–1100 (1999).

4. Borràs, M., Armengol, G., De Cabo, M., Barquinero, J.-F. & Barrios, L. Comparison of methods to quantify histone H2AX phosphorylation and its usefulness for prediction of radiosensitivity. *Int. J. Radiat. Biol.* **3002,** 1–10 (2015).

5. *ISCN (2013): An International System for Human Cytogenetic Nomenclature.* (S. Karger, 2013).

6. Armengol, G. *et al.* Genetic changes including gene copy number alterations and their relation to prognosis in childhood acute myeloid leukemia. *Leuk Lymphoma* **51,** 114–124 (2010).

7. Boissel, N. *et al.* Prognostic significance of FLT3 internal tandem repeat in patients with de novo acute myeloid leukemia treated with reinforced courses of chemotherapy. *Leukemia* **16,** 1699–1704 (2002).

8. González, J. R. *et al.* SNPassoc: An R package to perform whole genome association studies. *Bioinformatics* **23,** 644–645 (2007).

**SUPPLEMENTARY TABLES**

Supplementary Table S1. Clinical characteristics of the t-MN cases (n=45)

| Characteristic | n (%) |
| --- | --- |
| **Sex**  Female  Male | 22 (49%)  23 (51%) |
| **Primary diagnosis**  Hematologic malignancies  Solid tumors  Unknown | 25 (55%)  12 (27%)  8 (18%) |
| **t-MN**  t-AML  t-MDS | 28 (62%)  17 (38%) |
| **Cytogenetic features**  Loss of chromosome 5, 7, or both  t(11q23), t(21q22), t(15;17) or inv(16)  Normal karyotype  Other karyotypes  Unknown | 16 (35%)  8 (18%)  7 (16%)  12 (27%)  2 (4%) |
| **Age at t-MN diagnosis***, years | 54.47 (14-78) n=19 |
| **Latency***, months | 47.25 (11-180) n=16 |

*Values are median; minimum to maximum in parentheses

Supplementary Table S2. Genotype distribution for SNPs at *TP53* and *MDM2* in t-MN patients and controls.

| SNP and genotype | No. t-MN patients (%) | No. controls (%) |
| --- | --- | --- |
| *TP53* c.215G>C (Arg72Pro) |  |  |
| Arg/Arg | 19 (47.5) | 33 (61.1) |
| Arg/Pro | 15 (37.5) | 18 (33.3) |
| Pro/Pro | 6 (15) | 3 (5.6) |
| *MDM2* c.309T>G |  |  |
| T/T | 11 (28.2) | 28 (59.6) |
| T/G | 17 (43.6) | 14 (29.8) |
| G/G | 11 (28.2) | 5 (10.6) |

Supplementary Table S3. Mean γ-H2AX foci number per cell ± SEM in Jurkat p53Pro and Jurkat p53Arg cells after treatment with busulfan and doxorubicin

|  | Busulfan | | |  | Doxorubicin | | |
| --- | --- | --- | --- | --- | --- | --- | --- |
| Time | Jurkat p53Pro | Jurkat p53Arg | P value |  | Jurkat p53Pro | Jurkat p53Arg | P value |
| 0 h | 0.32±0.05 | 0.45±0.05 | 0.073 |  | 0.04±0.01 | 0.07±0.02 | 0.079 |
| 2.5 h | 13.65±0.54 | 21.05±1.07 | 0.000 |  | 0.45±0.07 | 0.20±0.05 | 0.121 |
| 5 h | 22.40±1.00 | 28.05±1.05 | 0.001 |  | 6.01±0.34 | 8.18±0.46 | 0.002 |
| 12 h | 20.57±1.36 | 34.51±1.68 | 0.000 |  | 39.08±1.34 | 43.80±1.40 | 0.026 |
| 24 h | 19.03±1.10 | 30.98±1.77 | 0.001 |  | 30.41±1.43 | 49.86±1.62 | 0.000 |
| 48 h | 17.85±0.56 | 19.20±0.96 | 0.909 |  | 24.44±0.90 | 23.28±1.35 | 0.532 |

**SUPPLEMENTARY FIGURES**


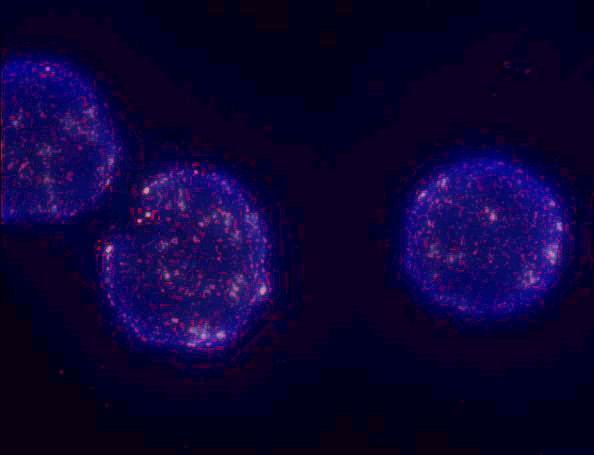


Figure S1. Representative image of γ-H2AX foci detected by immunofluorescence after drug treatment. Red dots indicate the foci and blue indicates nuclear counterstaining.


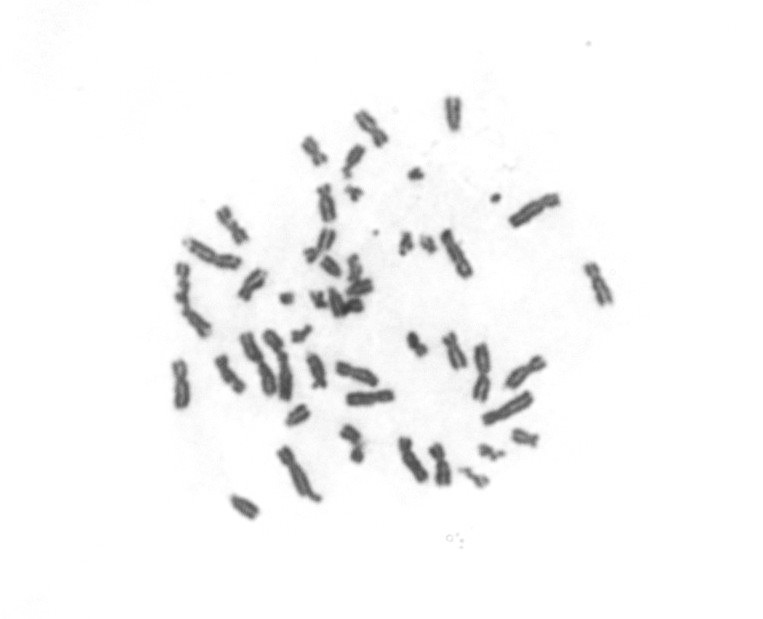


Figure S2. Representative image of chromosome breakage assay after drug treatment. A metaphase is shown with mutiple gaps and breaks.


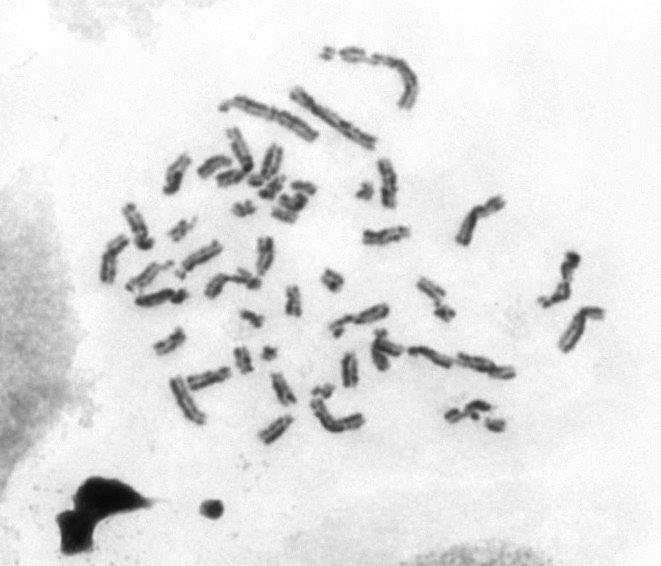


Figure S3. Representative image of sister chromatid exchange assay after drug treatment. A metaphase is shown with mutiple exchanges.


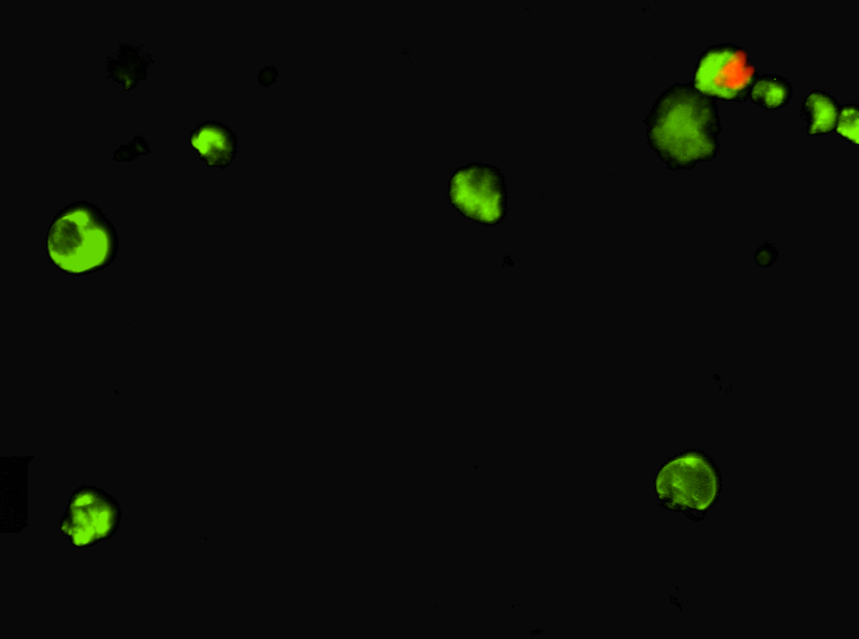


Figure S4. Representative image of apoptosis assay after drug treatment. Cells were stained with the Annexin-V-FLUOS Staining kit.
